# The Potency of an Anti-MERS Coronavirus Subunit Vaccine Depends on a Unique Combinatorial Adjuvant Formulation

**DOI:** 10.3390/vaccines8020251

**Published:** 2020-05-27

**Authors:** Parakkal Jovvian George, Wanbo Tai, Lanying Du, Sara Lustigman

**Affiliations:** 1 Laboratory of Molecular Parasitology, Lindsley F. Kimball Research Institute, New York Blood Center, New York, NY 10065, USA; jparakkal@nybc.org; 2 Laboratory of Viral Immunology, Lindsley F. Kimball Research Institute, New York Blood Center, New York, NY 10065, USA; WTai@nybc.org (W.T.); LDu@nybc.org (L.D.)

**Keywords:** adjuvants, adjuvant combination, aluminum, rASP-1, synergy, MERS-CoV, receptor-binding domain, functional antibody responses, T follicular helper cells, germinal center B cells

## Abstract

Vaccination is one of the most successful strategies to prevent human infectious diseases. Combinatorial adjuvants have gained increasing interest as they can stimulate multiple immune pathways and enhance the vaccine efficacy of subunit vaccines. We investigated the adjuvanticity of Aluminum (alum) in combination with rASP-1, a protein adjuvant, using the Middle East respiratory syndrome coronavirus MERS-CoV receptor-binding-domain (RBD) vaccine antigen. A highly enhanced anti-MERS-CoV neutralizing antibody response was induced when mice were immunized with rASP-1 and the alum-adjuvanted RBD vaccine in two separate injection sites as compared to mice immunized with RBD + rASP-1 + alum formulated into a single inoculum. The antibodies produced also significantly inhibited the binding of RBD to its cell-associated receptor. Moreover, immunization with rASP-1 co-administered with the alum-adjuvanted RBD vaccine in separate sites resulted in an enhanced frequency of TfH and GC B cells within the draining lymph nodes, both of which were positively associated with the titers of the neutralizing antibody response related to anti-MERS-CoV protective immunity. Our findings not only indicate that this unique combinatorial adjuvanted RBD vaccine regimen improved the immunogenicity of RBD, but also point to the importance of utilizing combinatorial adjuvants for the induction of synergistic protective immune responses.

## 1. Introduction

Vaccination is one of the most successful strategy to prevent infectious diseases in the human population, including those caused by emerging viruses [1]. Among various vaccine types, such as inactivated virus, live attenuated virus, and viral vector-based vaccines, subunit vaccines using proteins or peptides are believed to be much safer as they do not contain any live virus components and/or cause undesirable severe side effects [2]. However, unlike attenuated vaccines (composed of a virus or bacterium that replicates within the host) or inactivated vaccines (composed of either heat or chemically-inactivated parts of the pathogen), subunit vaccines (that are derived from known pathogen target antigens) are generally much less immunogenic, which can be improved with the addition of appropriate adjuvant(s) to the vaccine [1].

Adjuvants play an important role in enhancing the potency of subunit vaccines by improving humoral and/or cellular immune responses to the various subunit protein vaccines, decreasing the antigen dosages, and/or reducing immunization regimens [3,4]. Aluminum salts (hereinafter alum) are the most widely used adjuvants in licensed human vaccines, such as hepatitis A virus (HAV), HBV, human papilloma virus (HPV), and tetanus [1]. Alum has strong safety profiles, and generally stimulates humoral immunity through Th2-type immune responses, but it is a poor inducer of Th1-type humoral and cellular immune responses [1,5]. Two types of alum adjuvants are commercially available, including aluminum hydroxide and aluminum phosphate adjuvants [6]. Since alum induces a weak or no Th1 response, an increasing number of licensed human vaccines or those under clinical development have combined alum with other immunostimulatory molecules that induce different immune mechanisms and thus generate synergistic Th1- and Th2-type of responses [6]. For instance, AS04 adjuvant combines alum and the toll-like receptor 4 (TLR4) agonist monophosphoryl lipid A (MPL), and when used as an combinatorial adjuvant, it improves both humoral and cell-mediated immunity induced by the HBV and HPV vaccines [1,7]. Alum has also been combined with other immunostimulatory molecules, such as CpG (TLR9), QS-21, or GLA (TLR4). However, each combination requires that the ratios of the immunostimulant adjuvant to alum, as well as the sequence of mixing, are optimized in order for the adjuvanted vaccine to stimulate optimal protective immune responses [6].

The rASP-1 recombinant protein adjuvant corresponds to the *Onchocerca volvulus* secreted protein *Ov*-ASP-1. In previous studies, we have shown that rASP-1 is an immunopotentiating adjuvant that binds and activates innate cells [8,9,10], induces in mice a balanced Th1 (IgG2) and Th2 (IgG1)-associated antibody response, a biased Th1 cytokine response, and/or protection when combined with commercial vaccines (Haemorrhagic fever with renal syndrome, Influenza and Rabies), or with various experimental vaccine antigens such as ovalbumin, HIV-1 polypeptide, and the receptor binding protein domain (RBD) of severe acute respiratory syndrome coronavirus (SARS-CoV) spike (S) protein [9,10,11,12]. When used with the commercially available trivalent influenza vaccine (IIV3), in addition to protecting mice from lethal H1N1 challenge after one immunization with rASP-1-adjuvnated IIV3 vaccine, rASP-1 also facilitated antigen sparing of the IIV3 dose by 10 to 40-fold [12].

Middle East respiratory syndrome (MERS) coronavirus (MERS-CoV) is an emerging zoonotic virus that was first reported in 2012 [13]. The virus first binds to a cellular receptor dipeptidyl peptidase 4 (DPP4) through the receptor-binding domain (RBD) present within the S1 subunit of the S protein, whereas the S2 subunit undergoes conformational changes and subsequently mediates virus and cell membrane fusion, thus enabling MERS-CoV to enter into target cells [14,15]. The MERS-CoV RBD protein was identified as an important immunogen and the target for protective immune responses against the virus, which could also be induced by using experimental adjuvanted MERS-CoV RBD vaccines [16,17]. In previous studies, we have demonstrated that Montanide ISA51- or MF59-adjuvanted MERS-CoV RBD protein fused with a Fc tag of the human IgG (MERS-RBD-Fc) elicited strong antibody responses with neutralizing activity, and protected immunized transgenic mice against MERS-CoV infection [18,19]. In addition, a foldon (Fd) trimeric motif-fused MERS-RBD-Fd protein formulated with MF59 elicited long-term neutralizing antibodies with protective efficacy against MERS-CoV challenge in mice [20]. These data confirmed the potency of the MERS-RBD protein as a vaccine antigen against MERS-CoV infections.

There has been an increase interest in employing combinatorial adjuvants in a vaccine to improve the immunogenicity and potency of subunit, attenuated and anti-cancer vaccines [21,22,23]. Although alum has been tested for its combinatorial effects with various traditional immunostimulatory adjuvants, such as MPL, CpG, GLA, and QS21 [1,4], its combinatorial effect with a protein adjuvant, such as rASP-1 has not been investigated yet. In this study, we evaluated the adjuvanticity of alum (Th2-type stimulator) in combination with our novel rASP-1 protein adjuvant (Th1-type stimulator) using MERS-RBD-Fd (hereinafter RBD) as the model vaccine antigen. Notably, mice immunized with the combinatorial adjuvant system, where rASP-1 and the alum-adjuvanted RBD vaccine were co-administered separately, elicited neutralizing antibody titers that were similar to, or slightly lower than, those induced in mice immunized with either Montanide ISA51-adjuvanted or MF59-adjuvanted MERS CoV-RBD vaccines [18,19,20]. Notably, the functional antibodies induced by the co-administered combinatorial alum and rASP-1 vaccine were significantly and positively associated with an increased frequency of T follicular helper (TfH) and germinal center B (GC B) cells within the draining lymph nodes of the immunized mice.

## 2. Materials and Methods

### 2.1. Mice

First, 6–8-week-old female C57BL/6 mice were purchased from Jackson Laboratory (Bar Harbor, ME, USA). All mice were maintained in an AAALAC approved barrier facility at the New York Blood Center (NYBC, New York, NY, USA) and allowed to acclimatize for at least one week in the animal facility prior to use. All protocols involving mice were conducted with the approval of the Institutional Animal Care and Use Committee (IACUC Protocol 255.15) at NYBC.

### 2.2. Preparation of Recombinant Proteins

Recombinant MERS-RBD-Fd, MERS-RBD-Fc, and MERS-S1 proteins were prepared as previously described [20]. Briefly, genes encoding residues 377–588 of MERS-CoV S protein and a C-terminal Fd trimeric motif and His_6_ tag (MERS-RBD-Fd) or a C-terminal Fc tag of human IgG (MERS-RBD-Fc) were constructed into the hIgG-Fc vector, whereas gene encoding residues 18-725 of MERS-CoV S protein containing a C-terminal His_6_ tag (MERS-S1) was inserted into the pJW4303 vector. The sequence-confirmed recombinant plasmids were transfected into 293T cells using the calcium phosphate method, and culture supernatants containing expressed MERS-CoV proteins were collected 72 h post-transfection. The His-tagged (for MERS-RBD-Fd and MERS-S1) and Fc-tagged (for MERS-RBD-Fc) proteins were purified using Ni-NTA Superflow (Qiagen, Hilden, Germany) and Protein A affinity chromatography (GE Healthcare, Chicago, IL, USA), respectively.

Recombinant *Ov*-ASP-1 (rASP-1) was expressed using the T7-promoter driven pET28a expression vector (Novagen, Merck KGaA, Darmstadt, Germany), and the sequence-confirmed recombinant plasmid was transformed into *E. coli* BL21(DE3). The rASP-1 protein with an N-terminal His_6_ tag was expressed after induction with 1 mM IPTG for 5 h. The resulting inclusion bodies were solubilized in 1% SDS and purified with IMAC as described [12,24]. The purified protein remained soluble in PBS, pH 7.4 containing 0.1% SDS. The endotoxin level was less than 11.41 EU/mg, as measured by Endosafe cartridge (Charles River, Houston, TX, USA). To make sure that the residual LPS within the 25 µg rASP-1 used per mouse for immunization did not influence the adjuvant specific outcomes in the experiments listed below, the protein was pre-treated with Polymyxin B sulfate (Sigma-Millipore, Burlington, MA, USA) for 60 min at RT before use [12].

### 2.3. Mouse Immunization and Sample Collection

C57BL/6 mice were immunized intramuscularly (i.m.) twice, three weeks apart, with MERS-RBD-Fd (5 µg; hereinafter RBD) formulated with or without alum (Alhydrogel^®^ 250 µg) (InvivoGen, San Diego, CA, USA), rASP-1 (25 µg), or with distinct adjuvant combinations. Inoculums were prepared in a final volume of 100 µL per mouse and 50 µL was injected in the caudal thigh muscle of each hind leg in the appropriate site as outlined in Table 1. Control mice were injected with PBS in 0.1% SDS, the buffer solution of rASP-1 and is referred to as naive mice hereafter. Complete adsorption of the RBD and rASP-1 proteins by alum was confirmed by SDS-gel electrophoresis of the unbound protein samples after absorption with alum for 30 min on a rotator at RT. Sera samples were collected 7 days post-2nd immunization for analyses of anti-MERS-CoV neutralizing antibody titers, inhibition of MERS-CoV RBD-DPP4 receptor binding, and MERS-CoV RBD-specific antibody responses using MERS-CoV S1 as a target. The draining lymph nodes from each hind leg per mouse were also recovered at day 7 post-2nd immunization for analyses of the various cell profiles within.

### 2.4. MERS Neutralization Assay

The neutralizing activity of sera from the immunized mice against MERS-CoV infection in vitro was carried out using our established pseudovirus neutralization assay [17,20]. Briefly, 293T cells were co-transfected with a plasmid encoding the S protein of MERS-CoV (strain EMC2012) and a plasmid encoding Env-defective, luciferase-expressing HIV-1 genome (pNL4-3.luc.RE). The supernatants containing MERS-CoV S expressing pseudovirus collected 72 h post-transfection were incubated with serially diluted mouse sera at 37 °C for 1 h. The virus-serum mixtures were then added into Huh-7 cells expressing the MERS-CoV receptor DPP4. The cells were refed with fresh medium 24 h later, and after 72 h, lysed using cell lysis buffer (Promega, Madison, WI, USA) before the supernatants were transferred into 96-well luminometer plates. After addition of luciferase substrate (Promega, Madison, WI, USA), the plates were measured for relative luciferase activity using Infinite 200 PRO Luminator (Tecan, Männedorf, Switzerland). Neutralizing activity was calculated using the CalcuSyn computer program [25] and is expressed as 50% pseudovirus neutralizing antibody titers (NT_50_).

### 2.5. Inhibition of Binding of the Middle East Respiratory Syndrome Coronavirus (MERS-CoV) RBD-Fc Protein to DPP4-Expressing Huh-7 Cells

Sera from immunized mice were tested for their ability to inhibit the binding of the recombinant MERS-RBD-Fc protein to the cell-associated hDPP4 receptor in Huh-7 cells using flow cytometry analysis [26]. Briefly, cells were incubated with the MERS-RBD-Fc protein (5 µg/mL) in the presence or absence of diluted mouse sera (1:50) for 30 min at room temperature. After three washes and staining with FITC-labeled goat anti-human IgG Fc secondary antibody (1:500, Thermo Fisher Scientific, Waltham, MA, USA) for 30 min at room temperature, the cells were measured for fluorescence in a flow cytometer (BD LSRFortessa 4 system). Mean fluorescence intensity (MFI) values of the FITC channel from cells incubated with MERS-RBD-Fc protein in the absence of diluted sera were treated as 100 percentage binding. Inhibition of binding was calculated as the percentage of reduced binding to hDPP4 receptor in the presence of diluted sera from the different immunization groups versus the maximum binding observed in the absence of sera.

### 2.6. ELISA

ELISA to measure MERS-CoV RBD-specific antibody responses in immunized mouse sera was performed with MERS-CoV S1 as the target antigen and as previously described with some modifications [17,20]. The MERS-CoV S1 subunit of the MERS-CoV S protein contains the RBD region of the virus. Briefly, 96-well ELISA plates were coated with MERS-CoV S1 (1 µg/mL) overnight at 4 °C, blocked with 2% fat-free milk in PBS containing tween-20 (PBST) at 37 °C for 2 h, and then washed with PBST 3 times. The plates were subsequently incubated at 37 °C for 1 h with serially diluted mouse sera, and horseradish peroxidase (HRP)-conjugated anti-mouse IgG (1:5000), IgG1 (1:5000), or IgG2c (1:2000) antibodies (Thermo Fisher Scientific, Waltham, MA, USA). The substrate 3,3′,5,5′-tetramethylbenzidine (Sigma-Aldrich, St. Louis, MO, USA) was added to the plates after additional washes, and the reaction was stopped by the addition of 1 N H_2_SO_4_. Absorbance at 450 nm was measured using an ELISA plate reader (Tecan, Männedorf, Switzerland). Endpoint titers were calculated as the reciprocal of the highest dilution of sera giving an optical density greater than the mean ± 3 times the standard deviation of sera from naïve mice.

### 2.7. Profile of Cells in the Lymph Nodes

Draining lymph nodes from each hind leg per mouse were harvested at day 7 post-2nd immunization. The lymph nodes were dissociated into single cell suspensions using a syringe plunger, then passed through a 70 µm cell strainer and resuspended in complete RPMI 1640 media containing 10% fetal bovine serum (R10). Subsequently, 2.5 × 10^6^ cells were washed and resuspended in fresh R10 media in a 96-well cell culture plate for flow cytometry staining. 1.5 × 10^6^ cells were stained with a cocktail of the following fluorescently labeled antibodies: CD45-AF700, CD11b-PE-Cy5, CD11c-BV711, Ly6C-PerCP, CD40-APC, CCR7-PE, CD80-BV650, CD86-BV421, Ly6G-PE-Cy7, and B220-BV605. While 1 × 10^6^ cells were stained with a cocktail of the following fluorescently labeled antibodies: CD45-AF700, CD4-PE-Cy7, CXCR5-BV605, PD-1-BV421, B220-BV650 and GL-7-AF647 (all from Biolegend, San Diego, CA, USA), and CD95-BV510 (BD Biosciences, Dublin, Ireland), in a brilliant violet cell stain buffer (BD Biosciences, Dublin, Ireland) for 20 min in the dark at room temperature. Cells were then washed, resuspended in cell stain buffer (BioLegend, San Diego, CA, USA), and the number of stained cells was acquired using BD LSRFortessa cell analyzer (BD Biosciences, Dublin, Ireland). The data were analyzed using Flowjo Software (Tree Star, Ashland, OR, USA). CD45^+^CD11c^-^Ly6C^+^ cells were identified as monocytes, CD45^+^CD11c^-^Ly6C^+^CD40^+^ cells were identified as activated monocytes, CD45^+^CD11c^-^Ly6C^+^CCR7^+^ cells were identified as migratory monocytes, CD45^+^CD4^+^ cells were identified as CD4^+^ T cells, CD45^+^CD4^+^CXCR5^+^PD-1^+^ cells were identified as TfH cells, CD45^+^B220^+^ cells were identified as B cells and CD45^+^B220^+^CD95^+^GL-7^+^ cells were identified as GC B cells.

### 2.8. Statistical Analysis

One-way ANOVA test with Tukey’s multiple comparison was used for statistical analysis using GraphPad Prism v6 (GraphPad, San Diego, CA, USA). Spearman correlation was performed to determine the association of the fold increase in the frequency of TfH and GC B cells with neutralizing antibody titers using GraphPad Prism v6 (GraphPad, San Diego, CA, USA). *p* < 0.05: *, *p* < 0.01: **, *p* < 0.001: ***, *p* < 0.0001: ****. ND: not detectable.

## 3. Results

### 3.1. Enhanced Antibody Responses and Neutralization against Middle East Respiratory Syndrome Coronavirus (MERS-CoV) Is Induced When Mice Are Immunized with rASP-1 and the Alum-Adjuvanted MERS-RBD-Fd Vaccine in Separate Injection Sites

To investigate whether rASP-1 in combination with alum enhances the humoral immune responses induced by the MRES-RBD-Fd (herein after RBD) vaccine, we immunized C57BL/6 mice twice. three weeks apart, using a formulation where rASP-1 and the RBD vaccine proteins were completely adsorbed to alum and then administered as a single inoculum (Table 1; Group 5). This adjuvanted vaccine was compared to that in which rASP-1 was co-administered with the alum-adjuvanted RBD vaccine as two inoculums and in two separate sites of the caudal thigh muscle (Table 1; Group 6). RBD formulated with either rASP-1 or alum alone, RBD alone, and PBS alone were included as controls.

**Figure 1:**
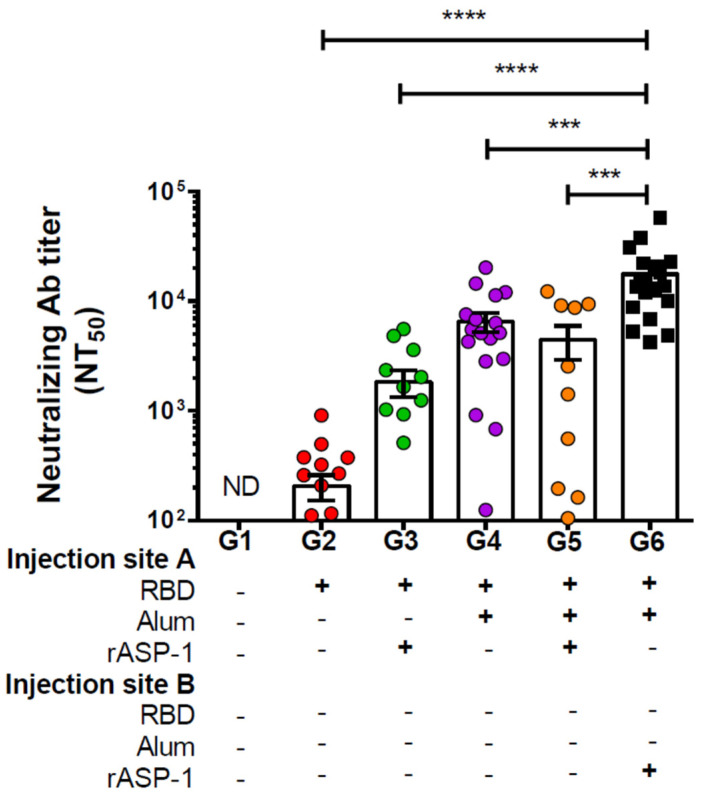
Neutralizing antibody titers against the pseudotyped Middle East Respiratory Syndrome Coronavirus (MERS-CoV) in sera of immunized mice: C57BL/6 mice were immunized i.m. with RBD with or without alum and rASP-1 alone or together using different combinations and/or formulations (Table 1 and X-axis legend). Sera samples were collected 7 days post-2nd immunization and analyzed for neutralization of the pseudotyped MERS-CoV. The data represents the mean and standard error (SEM) of NT_50_ titers from at least two independent experiments with 3 to 5 mice per group. “+” indicates the presence and “−“ indicates the absence of the protein or adjuvants in the formulation. Statistics was performed using one-way ANOVA with Tukey’s multiple comparison. *p* < 0.001: ***, *p* < 0.0001: ****, ND: not detectable.

Immunization of mice with rASP-1 and the alum-adjuvanted RBD vaccine in separate sites (G6, Figure 1) significantly resulted in the highest neutralizing antibody titers against MERS-CoV infection in vitro, NT_50_ = 17,657. It was approximately four-fold higher than in mice that were vaccinated with RBD + rASP-1 + alum in a single inoculum (G5 − NT_50_ = 4453; Figure 1), ~10-, ~3-, and 85-fold higher when compared to rASP-1-adjuvanted RBD vaccine, alum-adjuvanted RBD vaccine, and RBD only, respectively (G3 − NT_50_ = 1839, G4 − NT_50_ = 6528, G2 − NT_50_ = 207, respectively; Figure 1). It appears that this unique rASP-1 and alum combinatorial adjuvants promoted synergy in the functional humoral response produced vs. the vaccines that utilized the other formulations and/or regimens, including the rASP-1-adjuvanted RBD vaccine.

When the total IgG response to the MERS-RBD antigen was studied using the MERS-CoV S1 protein as the target protein, we found that although the RBD-specific total IgG antibody titers were ~8 times higher in mice that were vaccinated by co-administrating rASP-1 and the alum-adjuvanted RBD vaccine in separate sites (G6–142,525 end point titer), they were not significantly different from those elicited by immunization with RBD + rASP-1 + alum administered in a single inoculum (G5–18,149 end point titer), or with the alum-adjuvanted RBD vaccine (G4–53,104 end point titer; Figure S1A). Nevertheless, the co-administration of rASP-1 and the alum-adjuvanted RBD vaccine in separate sites significantly increased the RBD-specific total IgG antibody titers by ~20-fold compared to rASP-1-adjuvanted MERS-RBD vaccine or ~160-fold compared to the RBD vaccine alone (G6–142,525, G3–6700 and G2–872 end point titers respectively; Figure S1A); clearly showing that immunization using the unique rASP-1 and alum combinational adjuvants had a beneficiary effect in comparison to the rASP-1 (~20 fold) or the alum (~3 fold) adjuvanted vaccines.

To elucidate the IgG subtypes induced in the different immunization groups, we also analyzed the RBD-specific IgG1 and IgG2c antibody titers. We observed that the highest titer of IgG1 antibodies was induced when the RBD vaccine was formulated with alum alone or with rASP-1 + alum in one inoculum (G4–250,951 and G5–369,000 end point titers respectively; Figure S1B), with the titers being significantly higher when the vaccine was with both adjuvants and in one inoculum. The RBD-specific IgG1 titers were significantly decreased when rASP-1 was used as an adjuvant in aqueous formulations; when the rASP-1 and the alum-adjuvanted RBD were co-administered in separate sites (~7-fold decrease; G6–55,945 end point titers) or with the rASP-1-adjuvanted RBD vaccine (~40-fold decrease; G3–9165 end point titers) when it was compared to the administration of RBD + rASP-1 + alum in a single inoculum (G5–369,000 end point titers; Figure S1B). rASP-1 is known as an IgG2 (Th1)-biased adjuvant [8]. Notably, IgG2c responses were only elevated when rASP-1 was also administered as an adjuvant (G3–254, G5–1064, and G6–726 end point titers), with the combinatorial adjuvanted vaccines performing similarly and best (G5 and G6; Figure S1C).

These data suggest that when rASP-1 is adsorbed to alum in a vaccine formulation (G5), the two adjuvants work in synergy not only to elicit a stronger IgG1 response than the alum-adjuvanted vaccine formulation, but also for inducing the IgG2c antibody response as compared to alum-adjuvanted vaccine formulation alone (~14-fold increase), suggesting that the combination of rASP-1 and alum in a vaccine with the RBD antigen works in synergy to elicit a more balanced IgG1–IgG2c antibody response (IgG1/IgG2c ratio of 1626 in G5 vs. 4403 in G4; Figure S1D). The reduced IgG1/IgG2c ratio is more pronounced in a vaccine formulation where rASP-1 is not adsorbed to alum but co-administered separately (IgG1/IgG2c ratio of 601 in G6; Figure S1D).

### 3.2. Enhanced Inhibition of Binding of the MERS-RBD-Fc Protein to Cell-Associated hDPP4 Receptor Is Induced when Mice Are Immunized with rASP-1 and the Alum-Adjuvanted RBD Vaccine in Separate Injection Sites

**Figure 2:**
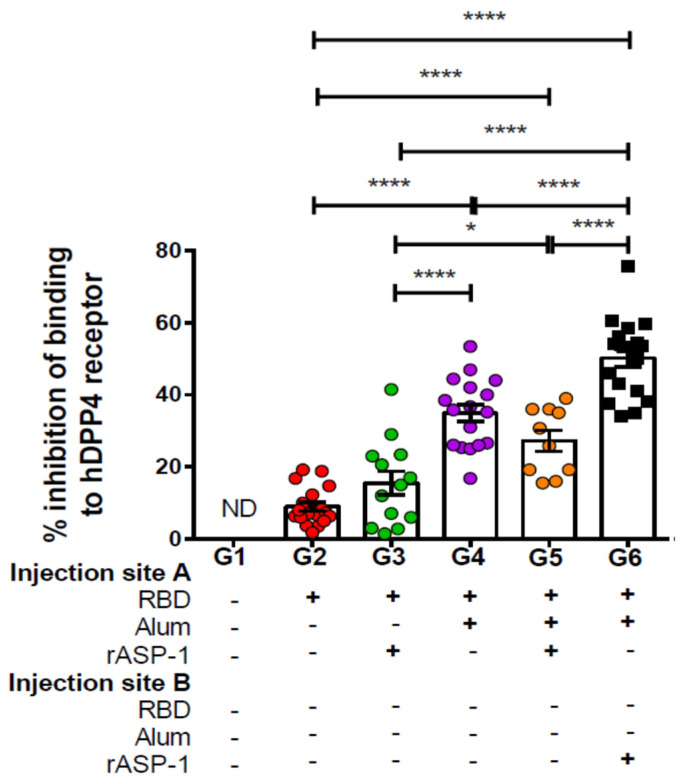
Inhibition of binding of Middle East Respiratory Syndrome Coronavirus (MERS-CoV) RBD-Fc to hDPP4 receptor by sera of immunized mice: C57BL/6 mice were immunized i.m. with MERS-RBD-Fd with or without alum and rASP-1 alone or together using different combinations and/or formulations (Table 1 and X-axis legend). Sera samples were collected on day 7 post-2nd immunization and assayed for inhibition of the binding of MERS-CoV RBD-Fc to Huh-7 cells expressing MERS-CoV receptor DPP4. The data represents the mean and standard error (SEM) of percentage inhibition of binding from at least two independent experiments with 3 to 5 mice per group. “+” indicates the presence and “−“ indicates the absence of the protein or adjuvants in the formulation. Statistics was performed using one-way ANOVA with Tukey’s multiple comparison. *p* < 0.05: *, *p* < 0.0001: ****. ND: not detectable.

Sera samples from day 7 post-2nd immunization were also tested for their ability to inhibit the binding of MERS-RBD-Fc protein to the hDPP4 receptor-expressing Huh-7 cells by flow cytometry. Similarly to the enhanced induction of neutralizing antibodies, immunization with rASP-1 and the alum-adjuvanted RBD vaccine in separate injection sites also increased the ability of the generated antibodies to inhibit the binding of the RBD protein to its receptor by ~2-fold (G6—50%; Figure 2) as compared to RBD + rASP-1 + alum adjuvanted vaccine in a single inoculum or with alum-adjuvanted RBD vaccine (G5–25% and G4–35%; Figure 2), and by >3-fold as compared to the rASP-1-adjuvanted RBD vaccine (G3–16%; Figure 2). When group G6 was compared to groups G3 and G4, a synergy of the combinatorial adjuvants, though when rASP-1 is not adsorbed to alum, was evident. However, when rASP-1 was adsorbed to alum (G5), the functionality of the antibodies elicited by the vaccine is much reduced. The inhibitory activity in group G5 was reduced by ~30%, albeit not significantly, as compared to G4.

### 3.3. Immunization with rASP-1 and the Alum-Adjuvanted RBD Vaccine in Separate Injection Sites Enhanced the Activation and Recruitment of Monocytes into the Draining Lymph Nodes

**Figure 3:**
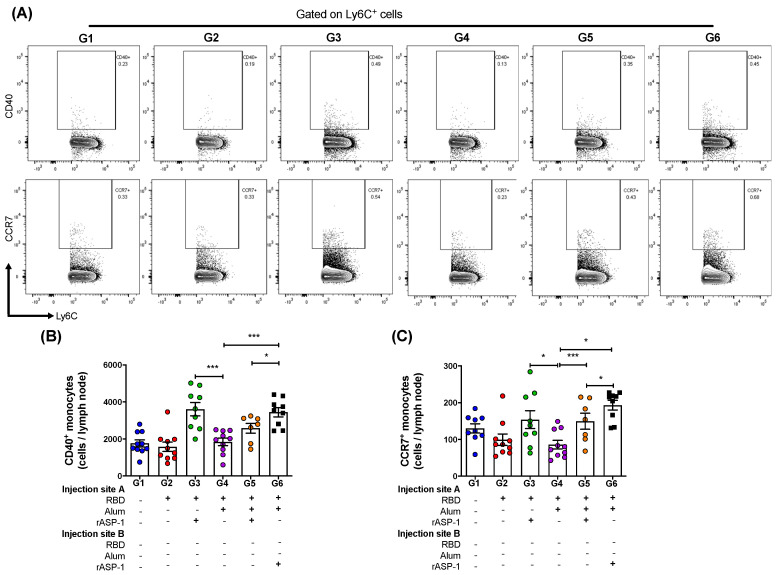
Monocyte subsets in the lymph nodes (LNs) of immunized mice: C57BL/6 mice were immunized i.m. with MERS-RBD-Fd with or without alum and rASP-1 alone or together using different combinations and/or formulations (Table 1 and X-axis legend). The draining LNs were harvested 7 days post-2nd immunization and the number of cells per LN were analyzed. **(A)** Representative flow cytometry plot of CD40^+^ monocytes (upper panel) and CCR7 ^+^ monocytes (lower panel in each immunization group. **(B)** The number of CD40^+^ monocytes, and **(C)** CCR7^+^ monocytes per LN were analyzed. The experiment was done once, and the data presented is from left and right draining LNs of 5 mice per group: mean and standard error. “+” indicates the presence and “−“ indicates the absence of the protein or adjuvants in the formulation. Statistics was performed using one-way ANOVA with Tukey’s multiple comparison. *p* < 0.05: *, *p* < 0.001: ***.

The draining lymph nodes (LN) were harvested from each leg 7 days post-2nd immunization and analyzed for the number of monocytes as well as their activation and migratory status. Migration of innate cells from the site of injection to the LNs are required to initiate an effective adaptive immune response [27]. The analyses demonstrated that there was no significant difference in the total number of monocytes (CD45^+^CD11c^-^Ly6C^+^; Figures S2A and S3A) recruited into the LN per mouse between the various immunization groups. However, the number of activated monocytes (C45^+^CD11c^-^Ly6C^+^CD40^+^) in the LN were significantly higher in mice where rASP-1 and the alum-adjuvanted RBD vaccine were co-administered in separate sites than in mice immunized with RBD + rASP-1 + alum in a single inoculum, or with alum-adjuvanted RBD vaccine (G6 vs. G5 and G4, respectively; Figure 3B). Notably, the number of activated monocytes in the LN of mice that received only the rASP-1-adjuvanted RBD vaccine (G3) were similar to those present in mice where rASP-1 was administered without being adsorbed to alum (G6). It appears that alum does not add to the activation of the monocytes in any of the formulations tested (G4, G5 or G6; Figure 3B).

Nevertheless, a synergistic effect was observed when rASP-1 was added to the RBD vaccine with or without alum with respect to CCR7^+^ (migratory) monocyte subset. The number of migratory monocytes (CD45^+^CD11c^-^Ly6C^+^CCR7^+^) in the LN were significantly higher in mice where rASP-1 and the alum-adjuvanted RBD vaccine were co-administered in separate sites, RBD + rASP-1 + alum was administered in a single inoculum or the rASP-1-adjuvanted RBD vaccine vs. the alum-adjuvanted RBD vaccine (G6, G5 and G3 vs. G4; Figure 3C). The number of migratory monocytes in the LN of mice that received the alum-adjuvanted RBD vaccine were actually similar to those in the control group that received only RBD (G4 vs. G2). As observed with the number of activated monocytes, migratory monocytes in the LNs of mice that received rASP-1 and the alum-adjuvanted RBD vaccine co-administered in separate sites were significantly higher in mice than in mice received RBD + rASP-1 + alum administered in a single inoculum (G6 vs G5; Figure 3C).

### 3.4. Immunization with rASP-1 and the Alum-Adjuvanted RBD Vaccine in Separate Injection Sites Enhanced the Frequency of TfH Helper and the GC B Cells, Both of Which Were Positively Associated with the Titers of the Functional Antibody Responses

**Figure 4:**
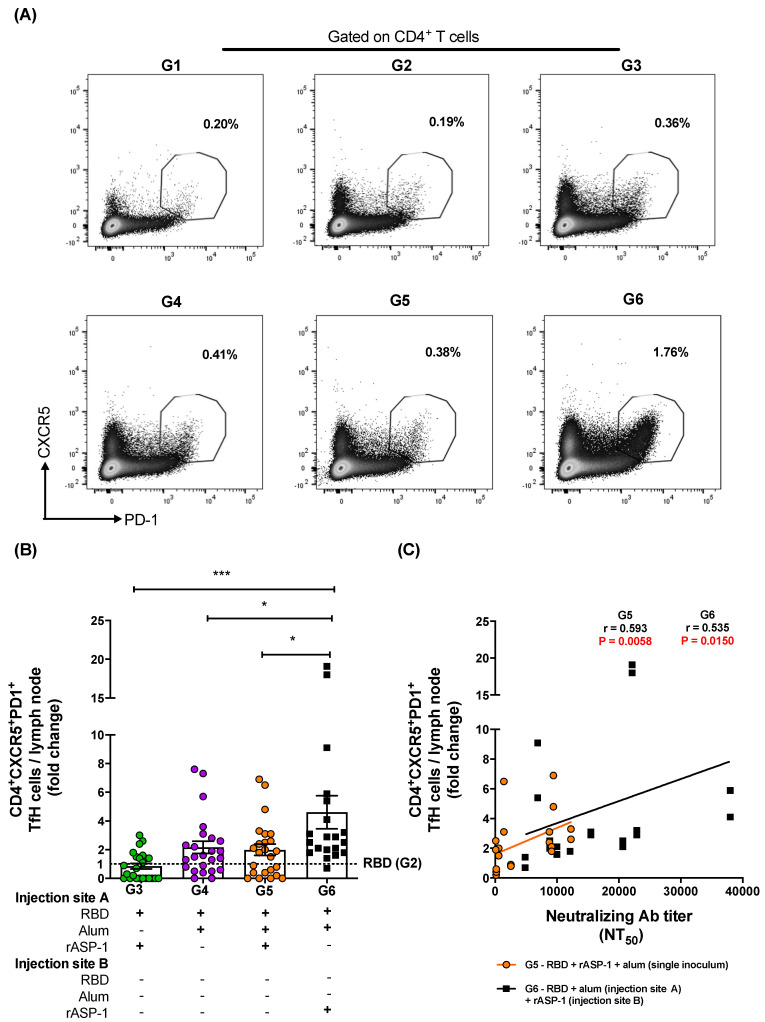
Fold increase in the frequency of TfH cells in the lymph nodes (LNs) of immunized mice: C57BL/6 mice were immunized i.m. with MERS-RBD-Fd with or without alum and rASP-1 alone or together using different combinations and/or formulations (Table 1 and X-axis legend). The draining LNs were harvested on day 7 post-2nd immunization and the frequency of TfH cells per LN was analyzed. **(A)** Representative flow cytometry plot in each immunization group. **(B)** The fold increase in the frequency of TfH cells per LN in all the adjuvanted-vaccine groups was normalized against the RBD vaccine group (G2). **(C)** The fold increase in the frequencies of TfH cells per LN from groups G5 and G6 were correlated with the NT_50_ titers determined in each serum sample of the corresponding mice. The experiment was repeated at least twice with 3 to 5 mice per group and the data are represented as mean and standard error of individual LN per mice. “+” indicates the presence and “−“ indicates the absence of the protein or adjuvants in the formulation. Statistics was performed using one-way ANOVA with Tukey’s multiple comparison. *p* < 0.05: *, *p* < 0.001: ***. Spearman correlation was performed to determine the association of TfH cells with neutralizing antibody titers.

**Figure 5:**
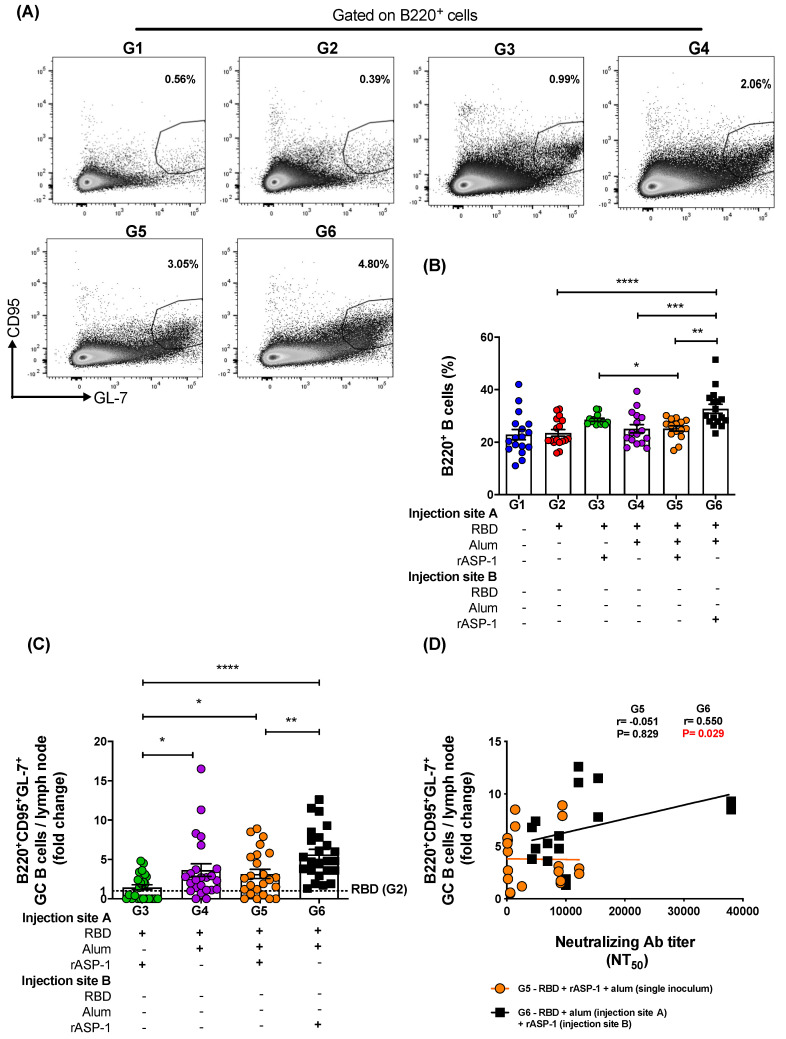
Frequency of B cells and fold increase in the frequency of GC B cells in the lymph nodes (LNs) of immunize mice: C57BL/6 mice were immunized i.m. with MERS-RBD-Fd with or without alum and rASP-1 alone or together using different combinations and/or formulations (Table 1 and X-axis legend). The draining LNs were harvested on day 7 post-2nd immunization and the frequency of GC B cells per LN was analyzed. **(A)** Representative flow cytometry plot in each immunization group. **(B)** The frequency B cells per LN as well as **(C)** the fold-increase in the frequency of GC B cells per LN was analyzed. The frequency of GC B cells per LN in all the adjuvanted-vaccine groups was normalized against the RBD vaccine group (G2). **(D)** The fold increase in the frequencies of GC B cells per LN in groups G5 and G6 were correlated with the NT_50_ titers determined in each serum sample of the corresponding mice. The experiment was repeated at least twice with 3 to 5 mice per group and the data are represented as mean and standard error. “+” indicates the presence and “−“ indicates the absence of the protein or adjuvants in the formulation. Statistics was performed using one-way ANOVA with Tukey’s multiple comparison. *p* < 0.05: *, *p* < 0.01: **, *p* < 0.001: ***, *p* < 0.0001: ****. Spearman correlation was performed to determine the association of GC B cells with neutralizing antibody titers.

To investigate the possible contribution of TfH and GC B cells to the robust functional antibody responses induced in mice administered with rASP-1 and the alum-adjuvanted RBD vaccine in separate injection sites, LN from 7 days post-2nd immunization were analyzed for the TfH (Figure 4A) and GC B (Figure 5A) cell frequencies. Although the frequency of total CD4^+^ T cells within the LN was not significantly different between the various immunization groups (Figure S4B), the frequency of the TfH (CD4^+^CXCR5^+^PD-1^+^) cells within the LN of mice administered with rASP-1 and the alum-adjuvanted RBD vaccine in separate sites (G6) was 2.3-fold higher than in mice administered with the RBD + rASP-1 + alum vaccine in a single inoculum, and 5.5- and 2.1-fold higher versus rASP-1- and alum-adjuvanted RBD vaccines (G6 vs. G3 and G4, respectively; Figure 4B).

Importantly, the fold increase in the TfH cells was positively and significantly associated with the neutralizing antibody titers against the pseudotyped MERS-CoV in two immunization groups, namely mice that were immunized with rASP-1 and the alum-adjuvanted RBD vaccine in separate sites (G6; *r* = 0.535, *p* = 0.0150) and mice immunized with RBD + rASP-1 + alum vaccine in a single inoculum (G5; *r* = 0.593, *p* = 0.0058) (Figure 4C).

Notably, the frequency of B cells (B220^+^) in the LN was also significantly higher in mice administered with rASP-1 and the alum-adjuvanted RBD vaccine in separate sites than in mice administered with RBD + rASP-1 + alum in a single inoculum, or with the alum-adjuvanted RBD vaccine or with the RBD vaccine alone (G6—33% vs. G5—25%, G4—25%, and G2—24% respectively; Figure 5B). Additionally, the frequency of B cells in mice immunized with rASP-1-adjuvanted RBD was also significantly higher as compared to mice immunized with RBD + rASP-1 + alum in a single inoculum (G3—29% vs. G5—25% respectively; Figure 5B). Formulating the vaccine with rASP-1 in an aqueous formulation (not adsorbed to alum) might have been critical for the increased number of B cells in these two vaccine formulations (G6 and G3).

When the frequency of GC B cells (B220^+^CD95^+^GL-7^+^) in the draining LN (Figure 5C) were analyzed, it appeared that immunization of mice with rASP-1 and the alum-adjuvanted RBD vaccine in separate sites (G6) induced ~2-fold increase in the frequency of the GC B cells versus immunization with RBD + rASP-1 + alum in a single inoculum and the alum-adjuvanted RBD vaccine (G5 and G4), and ~4-fold increase in GC B cells was induced versus immunization with the rASP-1-adjuvanted RBD vaccine (G3; Figure 5C). Interestingly, the alum-adjuvanted vaccines (G5 and G4) had >2-fold increase in GC B cells in the LN compared to rASP-1-adjuvanted RBD vaccine (G3; Figure 5C). Importantly, only when rASP-1 and the alum-adjuvanted RBD vaccine were co-administered in separate sites (G6) was the fold increase in the GC B cells positively and significantly associated with the neutralizing antibody titers against the pseudotyped MERS-CoV (*r* = 0.550, *p* = 0.029). No significant association was observed in mice when the RBD + rASP-1 + alum vaccine was administered (G5) as a single inoculum (*r* = −0.051, *p* = 0.829; Figure 5D).

## 4. Discussion

Adjuvants are essential components in both prophylactic and therapeutic vaccines since they ameliorate antigen-specific protective immune responses [28]. However, choosing the appropriate adjuvant that can be employed safely and that enhances vaccine efficacy is still elusive and needs to be optimized experimentally first [29].

Besides a handful of adjuvants such as CPG, Poly I:C, MPLA and MF59, aluminum-based (alum) adjuvants are being used in most of the adjuvanted vaccines for humans globally even today since its inception 85 years ago [30]. Although beneficial effects of alum as an adjuvant were observed with the DTap, HepB, and HepA vaccines, a biased Th2-type immune response, absence of strong cellular responses, and the induction of adverse reactions were some of the limitations found with the various alum-adjuvanted vaccines [30]. Therefore, the utilization of a combination of adjuvants in vaccines that can improve the safety and efficacy of vaccines against emerging pathogens is being actively pursued by the research community [31]. The use of combinatorial adjuvant system is beneficial since they can be tailored to target varied pattern-recognition receptors (PRRs) with each being able to enhance antigen-specific responses (cellular and humoral) in a complementary or synergistic outcome [32]. For instance, intranasal vaccination with emulsified fine particles like PELC in combination with LD-indolicidin enhanced protective influenza-specific serological immunity in mice [33]. MPL and CpG combination adjuvants promoted homologous and heterosubtypic cross protection when used with the inactivated split influenza virus vaccine [34]. The co-administration of alum and a TLR-7 adjuvant enhanced memory B cell response to lymphocytic choriomeningitis virus (LCMV) antigen [35]. Alum in combination with MPLA-HA-adjuvanted HBsAg increased both the magnitude and the persistence of HBsAg-specific immune responses against hepatitis B virus infection [36].

The aim of the present study was to explore the synergistic potential of combining the *O. volvulus*-derived protein adjuvant, rASP-1 with alum as a novel combinatorial adjuvant system using MERS-RBD-Fd as the model vaccine antigen. We have previously shown that rASP-1 enhances the immune response when co-administered in an aqueous formulation with several bystander vaccine antigens [9,10,11]. Moreover, we have also reported that rASP-1-adjuvanted trivalent influenza vaccine (IIV3) elicits a balanced IgG1/IgG2c response to IIV3 and protects mice following H1N1 virus challenge, potentially via MyD88-independent TLR4 signaling [8,12].

In this study, we have shown that mice immunized with RBD + rASP-1 + alum in a single inoculum elicited neutralizing antibody titers against pseudotyped MERS-CoV that were not significantly different from mice that received either rASP-1-adjuvanted RBD vaccine or alum-adjuvanted RBD vaccine alone (Figure 1). Notably, when rASP-1 and the alum-adjuvanted RBD vaccine were co-administered in separate sites the vaccine ameliorated the production of neutralizing antibody titers by ~4-fold as compared to the combinatorial adjuvant system administered in a single inoculum (Figure 1).

We also observed that mice that received two immunizations at three-week intervals of the combinatorial adjuvant system where rASP-1 and the alum-adjuvanted RBD vaccine were co-administered separately elicited neutralizing antibody titers against pseudotyped MERS-CoV infection that were similar to, or slightly lower than, those elicited in mice that received three immunizations of the Montanide ISA51-adjuvanted, or two immunizations of the MF59-adjuvanted MERS CoV-RBD-Fc or MERS-RBD-Fd vaccines [20]. Another noteworthy observation is that the heightened neutralizing antibody titers induced by the unique experimental combinatorial adjuvant system was achieved using 5 µg of the MERS-RBD-Fd vaccine protein, compared to 10 µg of MERS-RBD-Fc or MERS-RBD-Fd proteins used in previous studies [20]. This suggests that the rASP-1 in this unique combinatorial adjuvant system enabled also RBD dose sparing and with two immunizations. We have previously reported that rASP-1 also facilitates IIV3 antigen dose sparing up to a 10- or 40-fold decrease, and with a single immunization of the rASP-1-adjuvanted IIV3, mice were still protected from a lethal H1N1 influenza virus challenge [12].

Importantly, the antibodies elicited by the different combinatorial RBD-adjuvanted vaccines were also functional in their ability to inhibit the binding of MERS-RBD to the human DPP4 receptor. Mice that received rASP-1 and the alum-adjuvanted RBD vaccine separately inhibited the binding by ~2-fold more as compared to mice administered with RBD rASP-1 + alum in a single inoculum (Figure 2). Although mice that received alum-adjuvanted RBD vaccine significantly inhibited binding (35% ± 2.3) compared to rASP-1-adjuvanted RBD vaccine, they were not significantly different compared to the combinatorial adjuvant system administered in a single inoculum (Figure 2). The inhibition of binding, however, was only enhanced in the combinatorial adjuvant system where rASP-1 and alum-adjuvanted RBD vaccine are co-administered separately. These data collectively suggest that adsorption of rASP-1 to alum in a combinatorial adjuvant system does not enhance the functional antibody responses elicited by alum-adjuvanted RBD vaccine. While rASP-1 when not adsorbed to alum in a combinatorial adjuvant system was able to ameliorate the functional antibody responses. An important concern raised when anti-viral vaccine are developed, especially with the ongoing COVID-19 crisis, is that some vaccine approaches may induce unwantedly adverse side effects due to antibody dependent enhancement (ADE) and thus more severe pathology [37]. Since ADE is generally correlated to the neutralizing antibody titers, studies have also shown that high neutralizing antibody titer may eliminate the potential induction of ADE [38,39]. Previous studies on MERS-CoV have shown that MERS-CoV RBD-inducing neutralizing antibodies are positively correlated with protective immunity against MERS-CoV infection, and serum neutralizing antibody titers against live MERS-CoV infection of 1:40, 1:119, 1:141, or above (equivalent to NT_50_ neutralizing antibody titer of about 1:1,000 against pseudotyped MERS-CoV infection) are associated with a protective immune response with no evidence of immunological toxicity or eosinophilic immune enhancement [18,19,20,40]. In our study, we show immunization of mice with rASP-1 and the alum-adjuvanted MERS-CoV RBD vaccine in separate sites have induced NT_50_ neutralizing antibody titers greater than 1:15,000 against pseudotyped MERS-CoV infection. We expect that such high-titer neutralizing may prevent MERS-CoV infection in vivo without causing any adverse effects. However, this will have to be proven experimentally in the future.

In this study, we also observed that immunization with the alum-adjuvanted RBD vaccine elicits an RBD-specific IgG1-biased response, while the rASP-1-adjuvanted RBD vaccine elicits a balanced RBD specific IgG1-IgG2c response (Figure S1B,C). Notably, in the combinatorial adjuvant system where rASP-1 and the alum-adjuvanted RBD vaccine are co-administered separately the balanced IgG1/IgG2c response (Figure S1D) was preserved, while mice that received the combinatorial adjuvant system in a single inoculum elicited an IgG1-biased response (Figure S1B,D). These results suggest that the presence of rASP-1 in an aqueous formulation shifts the dominant IgG1 response elicited by the alum-adjuvanted RBD vaccine to a balanced IgG1-IgG2c response and this was more pronounced when rASP-1 was not adsorbed to alum.

Interestingly, the administration of the combinatorial adjuvant system showed differences in the Ly6C^+^ activated monocyte but not in CD11c^+^ activated DC subsets (Figure S3). This may likely be due to the presence of rASP-1 in the vaccine, since we have previously shown that intra-muscular injection rASP-1 alone or the rASP-1-adjuvanted trivalent influenza (IIV3) vaccine elicited an increased recruitment of monocytes than DCs at the site of injection (24 h after injection) as compared to PBS control group or IIV3 alone [12]. In the present study, the administration of the combinatorial adjuvant system where rASP-1 was completely adsorbed to alum (single inoculum immunization group) significantly reduced the number of CD40^+^ (activated) monocytes and CCR7^+^ (migratory) monocytes to the draining LN compared to the administration of the combinatorial adjuvant system where rASP-1 was not adsorbed to alum (Figure 3B,C). Interestingly, the number of CD80^+^ monocytes in the LN was similar whether rASP-1 + alum + RBD were administered as a single inoculum or as a co-administered vaccine in two separate sites. However, both of these vaccine formulations as well as the rASP-1 adjuvanted-MERS-RBD vaccine resulted in significantly higher number of recruited CD80^+^ monocytes than the alum-adjuvanted RBD vaccine, suggesting that the presence of alum did not significantly alter the number of CD80^+^ monocytes recruited by the combinatorial rASP-1 and alum adjuvanted-MERS-RBD vaccines (Figure S3B).

Moreover, there was a 2-fold increase in the number of the activated monocytes and migratory monocytes recruited to the draining LN in mice that received the rASP-1-adjuvanted RBD vaccine when compared to the alum-adjuvanted RBD vaccine (Figure 3B,C). The number of migratory monocytes doubled in the draining LN of mice immunized with the combinatorial adjuvant system where RBD + rASP-1 + alum was administered in a single inoculum as compared to the alum-adjuvanted RBD vaccine alone. The number of migratory monocytes further increased in mice that received the combinatorial adjuvant system where rASP-1 and the alum-adjuvanted RBD vaccine were co-administered separately (Figure 3C). There were no significant differences observed in the number of activated and migratory DCs across all the immunization groups. Collectively, these data suggest that the rASP-1 in the combinatorial adjuvant system may play a significant role in the enhanced recruitment of monocyte subsets. This is supported with the data where the administration of the rASP-1-adjuvanted RBD vaccine also significantly increased the number of activated (CD40^+^) and migratory (CCR7^+^) monocytes in the draining LN compared to alum-adjuvanted RBD vaccine (Figure 3B,C). Also, rASP-1 and alum may work in synergy to improve the number of migratory monocytes in the draining LN compared to what alum could do alone.

One of the important events in the generation of an adaptive cellular response is the effective migration of innate cells to the lymph nodes to encounter naïve T cells, a process in which CCR7, a chemokine receptor, is known to play a dominant role [41]. In addition, the absence of CCR7 has been shown to affect the magnitude of protective responses against viral infections in mouse models [42,43]. Therefore, we suggest that rASP-1, when not adsorbed to alum in a vaccine formulation, may improve the effective recruitment of innate cells that lead to the induction of effector adaptive cellular responses.

TfH cells can determine humoral immunity that is also derived from GC B cells, and therefore both of these cell types have become an important aspect for rational designs of more effective vaccines, in particular those depending on functional antibodies for their efficacy [44,45]. To better understand what contributed to the improved elicitation of functional anti-MERS-CoV neutralizing antibodies, the frequencies of TfH (CD4^+^CXCR5^+^PD-1^+^) cells and GC B (B220^+^CD95^+^GL-7^+^) cells in the draining LN of immunized mice were analyzed. A two-fold increase in both TfH and GC B frequencies were induced when rASP-1 and the alum-adjuvanted RBD vaccine were co-administered in separate sites as compared to the combinatorial adjuvant system where RBD + rASP-1 + alum were administered in a single inoculum (Figure 4B and Figure 5C). While no significant difference was observed in the fold increase of the frequency of GC B cells in the LN of mice that were immunized with rASP-1 and the alum-adjuvanted RBD vaccine co-administered separately as compared to the alum-adjuvanted RBD vaccine, a six-fold increase was observed when this was compared to rASP-1-adjuvanted RBD vaccine alone (Figure 5C). These data suggest that the complete adsorption of rASP-1 to alum diminished not only the ability to induce migratory monocyte, but also the development of cells that are important for mounting an effective humoral response.

Importantly, we found a significant and positive correlation between the neutralizing antibody titers in sera of mice vaccinated with rASP-1 and the alum adjuvanted RBD vaccine separately and the fold increase in the frequency of TfH and GC B cells recruited in the draining LN (Figure 4C and Figure 5D). Interestingly, the fold increase in the frequency of TfH cells was also significantly and positively associated with the titers of neutralizing antibodies in mice that were immunized with the combinatorial adjuvant system administered in a single inoculum (RBD + rASP-1 + alum; Figure 4B), suggesting that the rASP-1 and alum may work in synergy.

## 5. Conclusions

Our study demonstrates that a unique combination of rASP-1 (a helminth-derived protein) protein adjuvant with alum and the MERS-RBD-Fd using the model vaccine antigen enhanced the protective immune responses to MERS-CoV, despite the fact that adjuvants have to be co-administered separately (where rASP-1 was not adsorbed to alum). Also, for the first time, we were able to determine that the TfH and GC B cells in the LNs in mice immunized with combinatorial adjuvanted-MERS-RBD vaccine were significantly and positively associated with the essential functional protective immune responses to MERS-CoV neutralizing antibodies. Further studies will be necessary, however, to elucidate the precise underlining mechanisms of this unique adjuvant combination of rASP-1 and alum. In our study, it appeared that the adsorption of rASP-1 to alum reduced the immunopotentiating activities of either rASP-1 or alum. As the potency of rASP-1 is highest when it is in an aqueous formulation, a better understanding of the targets of multiple immune pathways that are induced may also help us utilize the rASP-1 protein adjuvant in combination with other PRR agonists that can be used in aqueous formulations as adjuvants in novel combinatorial formulations. Such combinatorial adjuvants may be more advantageous with subunit vaccine models that generally are known to induce suboptimal protective immune responses alone and/or induce vaccine enhanced disease (VED) when used with the alum adjuvant [46].

## Figures and Tables

**Table 1:** Immunization of mice using different combinations and/or formulations of the vaccines and the site of injection: C57BL/6 mice were immunized intramuscularly (i.m.) with MERS-RBD-Fd (RBD) formulated with or without alum and/or rASP-1 alone or together in different combinations and/or formulations. Mice were immunized twice, 3 weeks apart according to the various G1–G6 experimental groups either at the front (A: 50 µL of inoculum) and/or the back (B: 50 µL of inoculum) of the caudal thigh muscle in each hind leg.

Groups	Injection Site A (50 µL Front Caudal Thigh Muscle)	Injection Site B (50 µL Back Caudal Thigh Muscle)
1	PBS	PBS
2	RBD	PBS
3	RBD + rASP-1	PBS
4	RBD + alum	PBS
5	RBD + rASP-1 + alum	PBS
6	RBD + alum	rASP-1

## Supplementary Materials

The following are available online at https://www.mdpi.com/2076-393X/8/2/251/s1, Figure S1: Induction of MERS-CoV-RBD specific IgG subtypes in sera of immunized mice, Figure S2: Representative flow cytometry plot determining the gating strategy of the immune cells recruited to the draining lymph nodes (LNs) of immunized mice, Figure S3: Number of monocyte and DC subsets recruited into the lymph nodes (LNs) of immunized mice, Figure S4: Frequency of CD4^+^ T cells in the lymph nodes (LNs) of immunized mice.
