# Supplementary material for: The Potency of an Anti-MERS Coronavirus Subunit Vaccine Depends on a Unique Combinatorial Adjuvant Formulation
Source: Vaccines (Basel). 2020 May 27;8(2):251. doi: 10.3390/vaccines8020251 (PMC7350031; doi:10.3390/vaccines8020251)
Supplement: Supplementary file 1 [file vaccines-08-00251-s001.pdf]

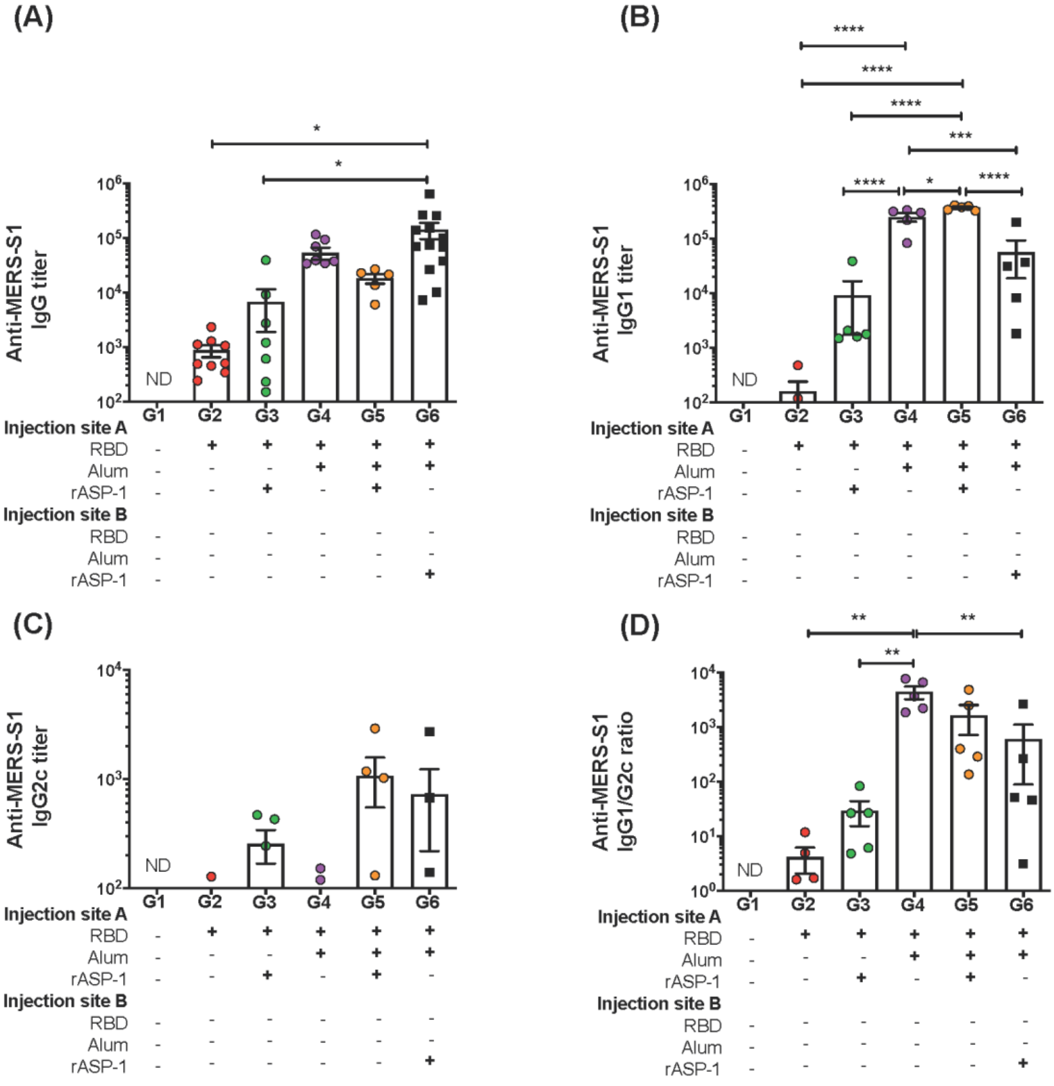

Figure S1. Induction of MERS-CoV-RBD specific IgG subtypes in sera of immunized mice: C57BL/6 mice were immunized i.m. with MERS-RBD-Fd with or without alum and rASP-1 alone or together using different combinations and/or formulations (Table 1 and X-axis legend). Sera samples were collected 7 days post-2nd immunization and assayed with MERS-CoV S1 as the target antigen for RBD-specific **(A)** IgG, **(B)** IgG1 and **(C)** IgG2c antibodies. **(D)** IgG1/G2c ratio. The data is from two independent experiments with 3 to 5 mice per group (for IgG) and a subset of 5 mice per group from one experiment for IgG1 and IgG2c. The data represent endpoint serum dilutions that remain positively detectable: mean and standard error. "+" indicates the presence and "−" indicates the absence of the protein or adjuvants in the formulation. Statistics was performed using one-way ANOVA with Tukey's multiple comparison. $p < 0.05$: *, $p < 0.01$: **, $p < 0.001$: ***, $p < 0.0001$: ****. ND: not

detectable,

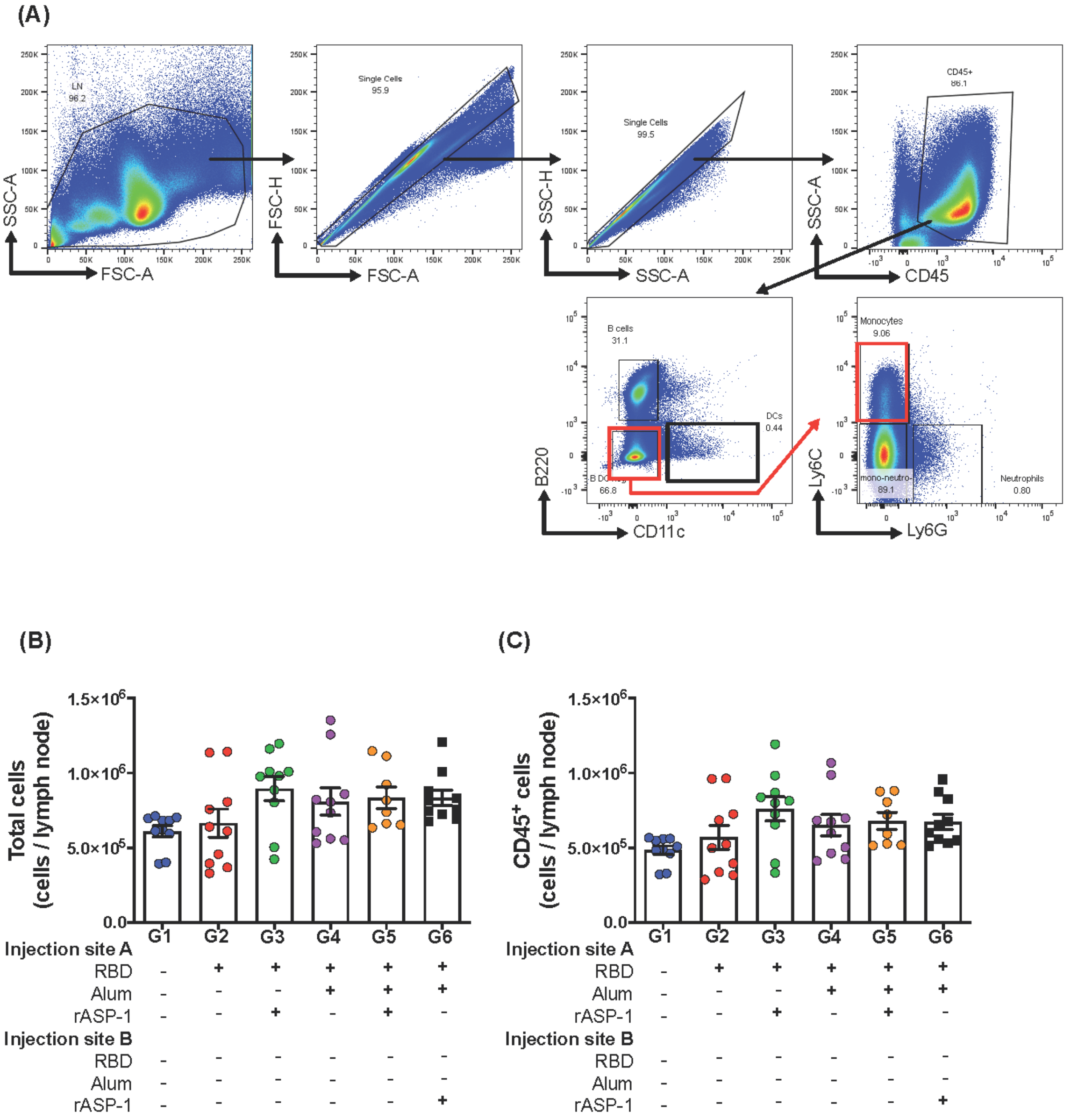

Figure S2: Representative flow cytometry plot determining the gating strategy of the immune cells recruited to the draining lymph nodes (LNs) of immunized mice: C57BL/6 mice were immunized i.m. with MERS-RBD-Fd with or without alum and rASP-1 alone or together using different combinations and/or formulations (Table 1 and X-axis legend). **(A)** Representative gating strategy of the immune cells recruited to the LNs. The draining lymph nodes were harvested 7 days post-2nd immunization and **(B)** the total number of cells and **(C)** CD45^{+} immune cells were analyzed per LN. The experiment was done once, and the data presented are from left and right draining LNs of 5 mice per group: mean and standard error. "+" indicates the presence and "−" indicates the absence of the protein or adjuvants in the formulation. Statistics was performed using one-way ANOVA

with Tukey's multiple comparison,

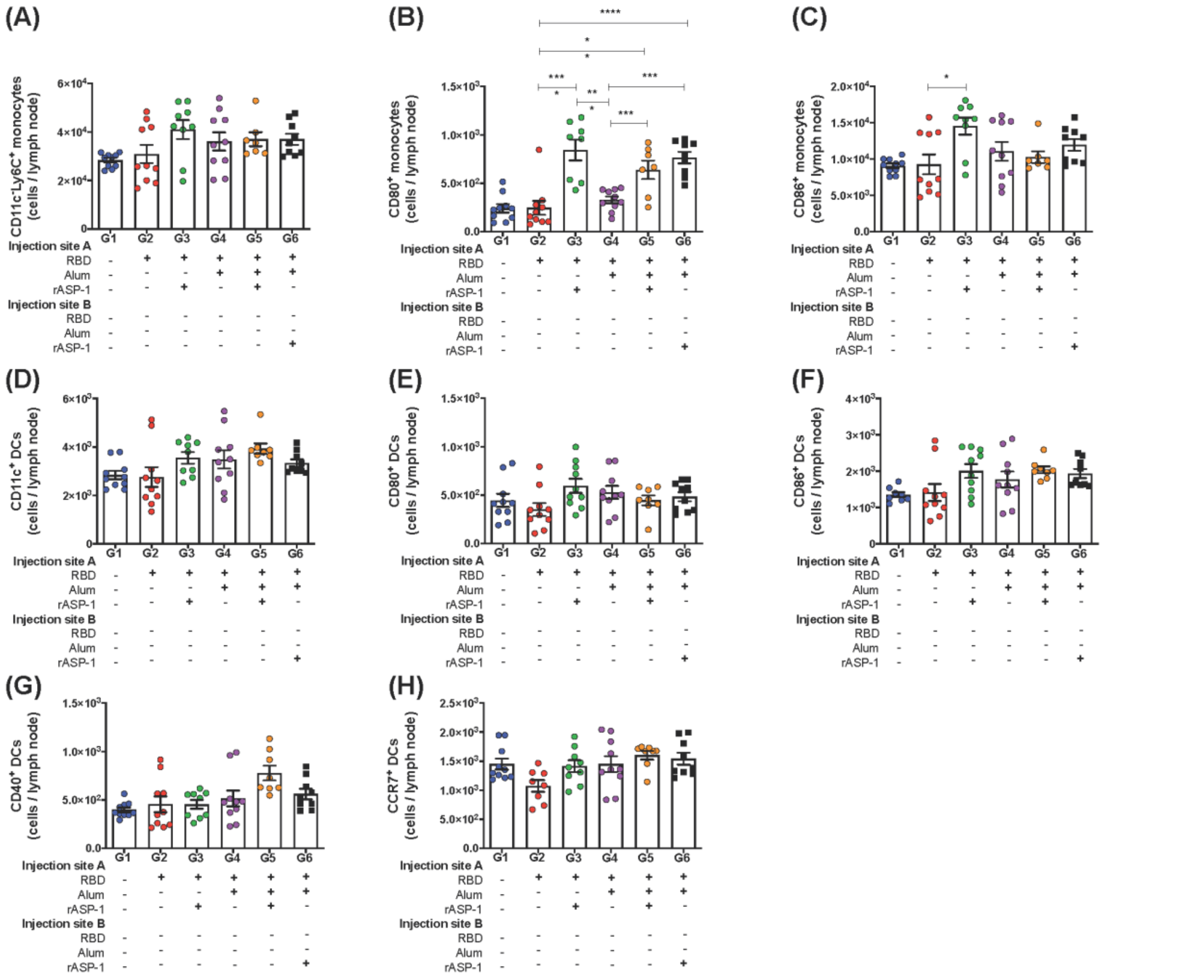

Figure S3: Number of monocyte and DC subsets recruited into the lymph nodes (LNs) of immunized mice: C57BL/6 mice were immunized i.m. with MERS-RBD-Fd with or without alum and rASP-1 alone or together using different combinations and/or formulations (Table 1 and X-axis legend). The draining lymph nodes were harvested 7 days post-2nd immunization and the number of **(A)** monocytes ($CD45^{+}CD11c^{-}Ly6C^{+}$), **(B)** $CD80^{+}$ monocytes, **(C)** $CD86^{+}$ monocytes, **(D)** DCs ($CD45^{+}CD11c^{+}$), **(E)** $CD80^{+}$ DCs, **(F)** $CD86^{+}$ DCs, **(G)** $CD40^{+}$ DCs, and **(H)** $CCR7^{+}$ migratory DCs were analyzed per LN. The experiment was done once, and the data presented is from left and right draining LNs of 5 mice per group: mean and standard error. "+" indicates the presence and "−" indicates the absence of the protein or adjuvants in the formulation. Statistics was performed using one-way ANOVA with Tukey's multiple comparison

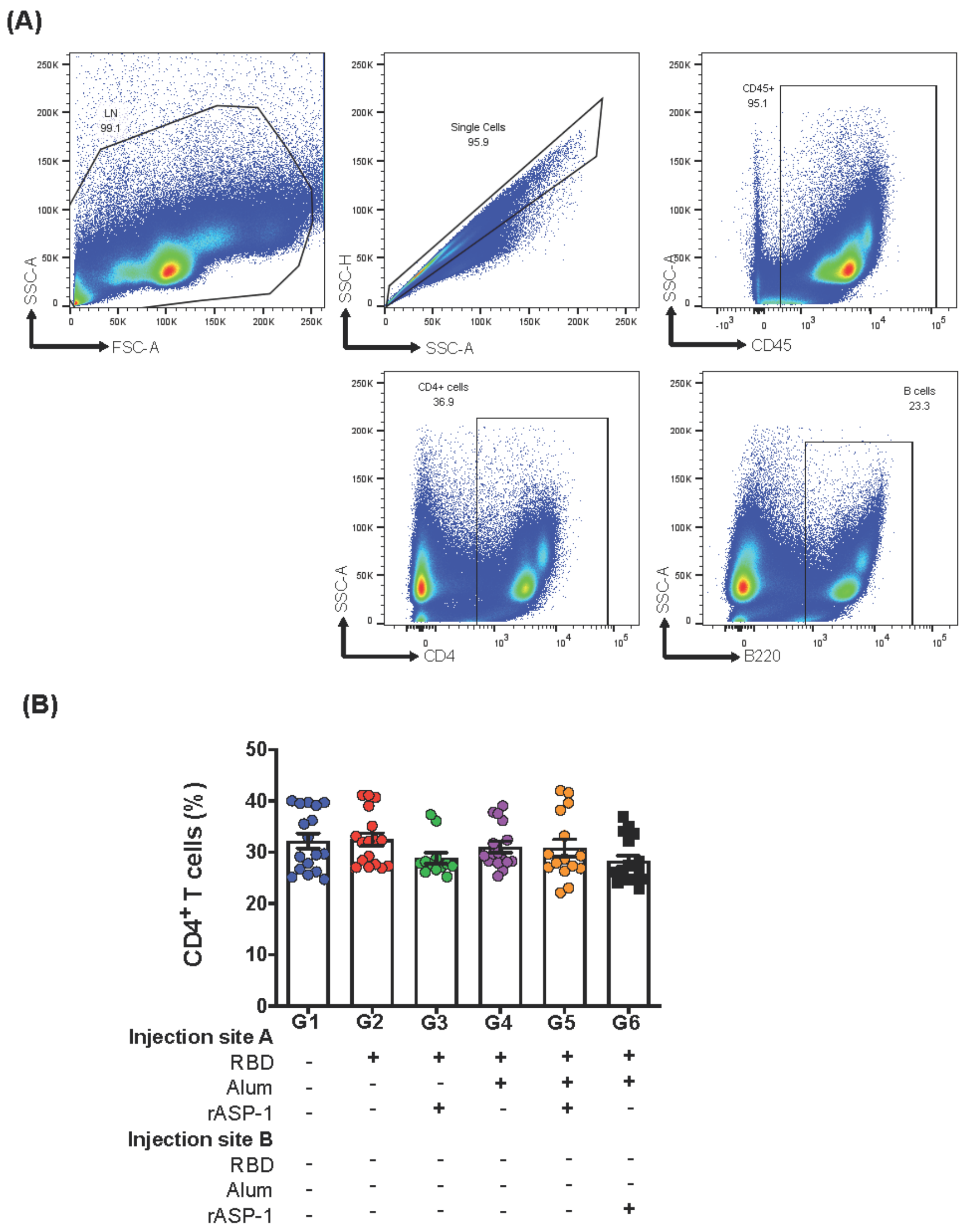

Figure S4. Frequency of CD4⁺ T cells in the lymph nodes (LNs) of immunized mice: C57BL/6 mice were immunized i.m. with MERS-RBD-Fd with or without alum and rASP-1 alone or together using different combinations and/or formulations (Table 1 and X-axis legend). The draining LNs were harvested 7 days post-2nd immunization. **(A)** Representative flow cytometry plot determining the gating strategy of $CD4^+$ T cells and $B220^+$ B cells per LN. **(B)** The frequency of $CD4^+$ T cells per LN were analyzed. "+" indicates the presence and "−" indicates the absence of the protein or adjuvants in the formulation. Statistics was performed using one-way ANOVA with Tukey's multiple comparison.
